# Nutrients Regulate the Effects of Arbuscular Mycorrhizal Fungi on the Growth and Reproduction of Cherry Tomato

**DOI:** 10.3389/fmicb.2022.843010

**Published:** 2022-04-08

**Authors:** Lei Wang, Xin Chen, Yeqin Du, Di Zhang, Zhanhui Tang

**Affiliations:** State Environmental Protection Key Laboratory of Wetland Ecology and Vegetation Restoration, School of Environment, Northeast Normal University, Changchun, China

**Keywords:** arbuscular mycorrhizal fungi, nutrient differences, growth and reproductive strategy, nutrient accumulation, cherry tomato

## Abstract

Arbuscular mycorrhizal fungi (AMF) colonize the rhizosphere of plants and form a symbiotic association with plants. Mycorrhizal symbionts have diversified ecological roles and functions which are affected by soil conditions. Understanding the effects of different AMF inoculation on plants under varied nutritional conditions is of great significance for further understanding the effects of the external environment regulating mycorrhizal symbiosis on plant phenotypic traits. In this study, the effects of four AMF inoculation treatments on the growth and reproductive performance of cherry tomato (*Solanum lycopersicum* var. *cerasiforme*) were investigated under three nutrient levels by pot experiment. It was found that the growth-promoting effect of AMF on cherry tomato decreased with nutrient reduction, and the effects of the same AMF inoculation treatment on cherry tomato were different at different nutrient levels. Nutrient levels and AMF had interactive effects on flower characteristics, fruit yield, resource allocation, and seed germination of the cherry tomato. In addition, AMF could promote sexual reproductive investment. Nutrient levels and AMF also affected the accumulation of nitrogen and phosphorus in cherry tomato, and there were significant differences among different AMF inoculation treatments. The results indicated that nutrient differences could affect the symbiosis between AMF and plants, and confirmed that there were differences in the effects of the four AMF inoculation treatments on the growth and reproductive traits of plants. The differences in growth and reproduction characteristics of cherry tomato between different AMF inoculation treatments at different nutrient levels indicated that the effects of AMF mycorrhizal on the traits of cherry tomato were regulated by nutrients.

## Introduction

Arbuscular mycorrhizal fungi (AMF) are ancient and widely distributed symbiotic fungi of plant roots. In terrestrial ecosystems, more than 80% of plants can form mycorrhizal symbiosis with AMF (Berruti et al., 2016; Genre et al., 2020). AMF plays an important role in the process of nutrient transfer between soil and plants. The common mycorrhizal networks (CMNs) formed by root peripheral hyphae is the main channel for nutrient exchange between aboveground and belowground in the ecosystem and plays an important regulatory role in the nutrient cycle of the ecosystem (Bever et al., 2010; Cheng et al., 2012; Johnson et al., 2013; Yang et al., 2021). AMF provides host plants with water and soil mineral nutrient resources (especially phosphate) in exchange for photosynthates and lipids needed for growth and reproduction (Jeffries et al., 2003; Jiang et al., 2017; Wang et al., 2017; He et al., 2019). This mutually beneficial symbiotic relationship of AMF and host plants can not only satisfy their growth needs but also improve the photosynthetic efficiency and promote the growth and development, which improve productivity and the biomass accumulation of host plants (Van Geel et al., 2016; Keymer and Gutjahr, 2018; Jajoo and Mathur, 2021). Mycorrhizal symbionts can also alleviate abiotic stresses such as drought and nutrient deficiency to the host plant (Hoeksema et al., 2010; Doubková et al., 2013; Nouri et al., 2014; Augé et al., 2015). The effects of AMF symbionts on plant growth vary with environmental conditions and may show promoting, inhibiting, or neutral effects on plant growth (Facelli et al., 2010). It is noteworthy that AMF can also affect the reproductive process of host plants to a certain extent; however, the role of AMF in regulating the flowering and reproduction of host plants is often ignored in most studies (Adeyemi et al., 2021; Carmen et al., 2021; Kazadi et al., 2021; Khaekhum et al., 2021; Chen et al., 2022). The positive effects of AMF on plants are widely recognized, but the effects of the AMF symbiosis with plant roots on growth and reproductive performance are extremely complex under different cultivation conditions which require further investigation.

The growth and reproduction traits of plants are plastic to the changes of environment, especially the changes of soil nutrients (Kattia et al., 2015). Therefore, the plants will change their growth and reproduction strategies in response to the changes in nutrients. As an important node between aboveground and belowground ecosystems, AMF is sensitive to soil nutrient changes (Averill et al., 2018; Pan et al., 2020). Host plants need to consume a large amount of energy to maintain the growth and reproduction of AMF, although AMF can provide host plants with nutrients needed for growth and development when plants are in an environment containing highly available nutrients (Parniske, 2008; Smith and Smith, 2011). Based on the ecological economics theory of trade partnership, it is predicted that the host plant may have a tradeoff strategy of reducing the degree of mycorrhizal symbiosis and thus inhibiting the growth promotion effect of AMF, which may lead to the transformation of mycorrhizal function from mutualism to commensalism or even parasitism (Floss et al., 2013; Ma et al., 2020; Pan et al., 2020). Host plants may reduce their dependence on AMF and thus reduce their photosynthate allocation to AMF when nutrients are abundant (Jiang et al., 2018; Verlinden et al., 2018). When plants grow in nutrient-deficient environments, abiotic stress usually stimulates the colonization of AMF in roots and affects mycorrhizal structures (e.g., arbuscular abundance and vesicles) (Martínez-García et al., 2012). The external environment of nutrient deficiency is conducive to the formation of mycorrhizal, promotes the symbiosis between AMF and plants, and improves the ability of plants to adapt to the adverse environment (Cosme and Wurst, 2013; Fusconi, 2014; Pozo et al., 2015). AMF can effectively alleviate phosphorus deficiency stress faced by host plants when they are in a phosphorus-deficient environment (Smith et al., 2004; Yang et al., 2012). Interestingly, the establishment of a symbiotic relationship between AMF and plants seems to be regulated only by soil nitrogen and phosphorus content, while other mineral elements in soil do not affect mycorrhizal formation (Landis et al., 2004; Nouri et al., 2014). Although soil nutrient levels and AMF can affect plant growth and reproduction, it is not clear how direct or indirect nutrient exchange between plants and AMF affects plant adaptation strategies under different nutrient levels, and whether there are differences in the effects of different AMF inoculation treatments under different nutrient levels.

The effect of mycorrhizal symbionts on plants may be affected by abiotic environmental factors, such as soil nutrient levels. Dwarf cherry tomato (*Solanum lycopersicum* var. *cerasiforme*) was selected as the plant material of this study because of its advantages of a short shoot, less space occupation, and short growth and reproduction cycle lasing 3–4 months (Rahim et al., 2019). We explored the effects of different nutrient levels and different AMF symbiosis and their interactions on the growth and reproductive strategies of cherry tomato, as well as the accumulation of nitrogen and phosphorus in cherry tomato. We hypothesized that (a) cherry tomato may become less dependent on AMF at a high nutrient level. However, with the decrease of nutrient level, the growth-promoting effect of AMF may be enhanced, and the promoting effect of different AMF inoculation treatments should be different; (b) AMF inoculation will change the reproductive strategy of cherry tomato at different nutrient levels. Especially at low nutrient levels, AMF may promote the absorption of nutrients and increase their investment in the reproduction of cherry tomato, and enhance the germination of offspring seeds; (c) different AMF treatments promoting/inhibiting the uptake and accumulation of nitrogen and phosphorus in cherry tomato under different nutrients. To test these hypotheses, we inoculated cherry tomatoes with four AMF treatments under three nutrient levels in the greenhouse, aiming to explore the response of growth and reproduction strategies of cherry tomato and nutrient accumulation to AMF treatments under different nutrient levels.

## Materials and Methods

### Seed Disinfection and Germination

The seeds of dwarf cherry tomato (*Solanum lycopersicum* var. *cerasiforme*) with full grains and consistent size were selected and soaked in 0.5% potassium permanganate solution for 5 min, 10% H_2_O_2_ for 10 min, and 5% NaClO_3_ for 1 min, respectively, for surface disinfection. The disinfected seeds were washed with pure water and then placed in petri dishes with moist filter paper. The petri dishes with seeds were placed in a constant temperature light incubator at 25°C (20% illumination and 60% humidity) for germination.

### Preparation of Soil Substrate

Using a mixture of peat soil and sand as soil substrate (Table 1), the following 3 nutrition treatments were set: high nutrition level [High, 1:1 (volume), peat soil: sand], medium nutrition level (Medium, 1:2, peat soil: sand), and low nutrition level (Low, 1:3, peat soil: sand). The prepared soil matrix was autoclaved at 126°C for 120 min, and then put into a flowerpot (inner diameter 16.5 cm, height 12.5 cm), wiped, and sterilized with 95% alcohol. A 2 L of soil matrix was added to each flowerpot.

**TABLE 1:** Basic characteristics of experimental soil substrate.

Properties	Value (Mean ± SE)	
	
	Peat soil	Sand
Density (kg/m^3^)	508.933 ± 12.639	1,462.400 ± 7.965
pH	4.905 ± 0.025	0
Organic matter, OM (%)	14.647 ± 0.165	0
Total nitrogen, TN (mg/kg)	4,796.343 ± 37.616	0
Available nitrogen, AN (mg/kg)	314.720 ± 17.638	0
Total phosphorus, TP (mg/kg)	6,231.953 ± 113.720	0
Available phosphorus, AP (mg/kg)	186.208 ± 2.407	0
Available potassium, AK (mg/kg)	4,169.227 ± 115.888	0
		

### Arbuscular Mycorrhizal Fungi Inoculation

Four AMF inoculation treatments were set up as follows: *Funnelliformis mosseae* (Fm), *Rhizophagus intraradices* (Ri), *Glomus versiforme* (Gv), and an equal mixture of the three fungi (Ma) (AMF strain information and propagation method in Supplementary Material). The dosage of the above treatments was 40 g (containing 1,000 spores), respectively. The control treatment (CK) without AMF inoculation was set at the same time. After wetting the soil with pure water, four fungus agent were added into each pot. To ensure the same microbial conditions except for AMF among different inoculation treatments, an equal number of bactericidal inoculums and 40 ml of water filtrate of non-sterilized microbial inoculums (filtered by a filter membrane with an aperture of 20 μm) were applied in the non-inoculation treatment (CK). One germinated seed was put on the fungus agent to ensure that the seeds can fully contact with the fungus agent after taking root, and then cover the germinated seeds and the fungus agent with a small amount of soil substrate. Finally, sufficient amount of pure water was added to make the whole soil substrate wet. There were 15 (3 nutrient levels × 5 inoculations including CK) treatment combinations in total, and each treatment combination was repeated 10 times for a total of 150 pots in this study.

### Plant Cultivation

All the pots with different treatments were placed in a greenhouse for cultivation with a photoperiod of 12 h/12 h (light/dark), an optical quantum flux density of 550–600 μmol/m^2^s, temperature of 30/25°C (day/night), and humidity of 40–50%. The plants were checked and watered every day to keep the soil moist. The positions of pots were randomly changed every week and the growth of the plants was monitored and recorded regularly until the cherry tomatoes were mature.

### Measurement

The flowering time was recorded after the first cherry tomato flower opened. Five plants were randomly selected from each treatment and the flower longevity was recorded. Blooming flowers with a petal opening angle of 180° were sampled to measure flower diameter and single flower weight (*n* = 5). The pollen number in single flowers was counted under an optical microscope after the crushed anthers were made into a suspension using 20% sodium hexametaphosphate solution (*n* = 5). Pollen viability of cherry tomato was determined by triphenyl tetrazole chloride method (Sutyemez, 2011) (*n* = 5). The number of flowers per plant was recorded every day until the plant was harvested (*n* = 10).

Fruit number and fresh weight were measured after harvest. Mycorrhizal colonization was measured by trill benzene blue staining (Brundrett et al., 1984). The stems, leaves, roots, and fruits of cherry tomato were placed in a drying oven at 80°C for 48 h. The biomass (the dry weight) of the stems, leaves, roots, and fruits were weighed, respectively, to calculate the root–shoot ratio and reproductive allocation (*n* = 10). The seeds were picked out after fresh cherry tomato fruits were crushed in water and record the number of seeds in each fruit (*n* = 5). Seed weight was weighed after drying in natural ventilation. These seeds were germinated after disinfection referring to the method in 2.1, and the seed germination percentage was calculated. The total contents of nitrogen and phosphorus in the aboveground and belowground parts of cherry tomato are determined by element analyzer (model: EA3100 Elemental Analyzer, Italy) after the plants were dried, crushed, and sieved (100 mesh) (*n* = 5).

### Data Statistics and Analysis

Before analysis, all data are checked to see whether they conform to normal distribution using the 1-Simple Kolmogorov–Smirnov *Z* test and variance homogeneity using the Bartlett test. All data conform to normal distribution and variance homogeneity. The effects of nutrients and AMF on the growth and reproduction of cherry tomato were tested by two-factor ANOVA. Multiple comparisons between treatments were performed using the least significant difference (LSD) test for differences at 0.05 significance level. All statistical analyses were performed using SPSS (25.0) (SPSS Inc., Chicago, IL, United States).

## Results

### Mycorrhizal Infection

**FIGURE 1:**
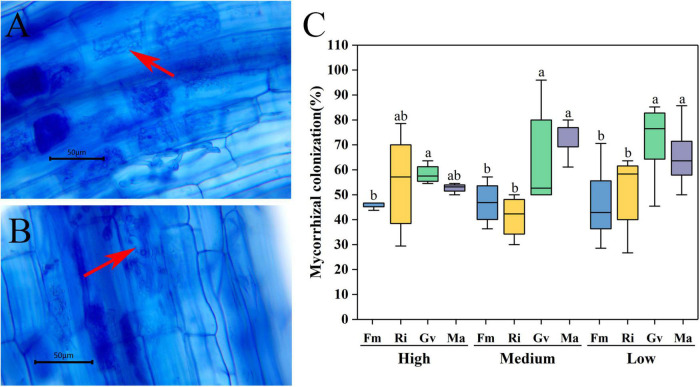
Mycorrhizal infection of cherry tomato root inoculated with arbuscular mycorrhizal fungi (AMF) at different nutrient levels. **(A,B)** Arbuscular structure of root (arrow); **(C)** Mycorrhizal colonization of cherry tomato under different nutrient levels. Different letters indicate that there were significant differences among different AMF inoculation treatments at the same nutrient level (*P* < 0.05), the same as below.

Arbuscules were formed in the root system of cherry tomato after AMF inoculation (Figures 1A,B), while no endophytic mycelia were found in the root system of cherry tomato without AMF inoculation. The root mycorrhizal infection of cherry tomato showed an extremely significant difference among different AMF inoculation treatments (*P* = 0.001). Soil nutrient level and the interaction between nutrients and AMF inoculation did not significantly affect the root infection rate (Table 2). Under the same nutrient levels, the infection rate of different AMF was also different. Overall, the infection rates of Gv and Ma were significantly higher than Fm and Ri at medium and low nutrient levels, and the infection rates of Gv were higher than Fm at a high nutrient level (Figure 1C).

**TABLE 2:** Effects of nutrient levels, AMF inoculation treatments and their interaction on root infection rate, growth and reproductive traits of cherry tomato.

Indicators	Nutrient levels (*df* = 2)		AMF (*df* = 4)		Nutrient levels *AMF (*df* = 8)	
			
	*F*	*P*	*F*	*P*	*F*	*P*
Mycorrhizal colonization rate	0.71	0.497	6.55	**0.001**	1.16	0.343
Aboveground biomass	176.82	**<0.001**	4.65	**0.002**	1.71	0.102
Belowground biomass	24.03	**<0.001**	3.16	**0.016**	0.94	0.490
Total biomass	165.16	**<0.001**	4.46	**0.002**	2.14	**0.036**
Root-shoot ratio	2.93	0.057	0.37	**0.012**	0.99	0.446
Flowering time	2.55	0.082	5.89	**<0.001**	5.15	**<0.001**
Flower longevity	1.07	0.353	2.32	0.071	1.41	0.218
Flower diameter	3.50	**0.033**	0.69	0.602	2.36	**0.022**
Flower weight	21.89	**<0.001**	1.55	0.193	2.77	**0.008**
Pollen number	0.91	0.408	0.55	0.701	0.75	0.646
Pollen viability	1.98	0.149	3.89	**0.008**	0.38	0.928
Flower number	14.01	**<0.001**	5.33	**0.001**	4.62	**<0.001**
Fruit number	3.81	**0.025**	2.88	**0.026**	3.81	**0.025**
Fruit biomass	22.20	**<0.001**	10.94	**<0.001**	2.33	**0.027**
Reproductive allocation	3.60	**0.032**	7.78	**<0.001**	3.14	**0.004**
Seeds in a fruit	4.76	**0.012**	2.74	**0.036**	1.31	0.254
1000-grain seed weight	7.63	**0.002**	1.15	0.351	1.45	0.216
Germination percentage	10.07	**<0.001**	5.00	**0.003**	3.80	**0.003**
Aboveground nitrogen accumulation	566.15	**<0.001**	21.95	**<0.001**	6.07	**<0.001**
Belowground nitrogen accumulation	324.87	**<0.001**	36.99	**<0.001**	7.76	**<0.001**
Total nitrogen accumulation	833.42	**<0.001**	37.44	**<0.001**	8.06	**<0.001**
Aboveground phosphorus accumulation	1360.17	**<0.001**	42.47	**<0.001**	2.41	**0.038**
Belowground phosphorus accumulation	188.94	**<0.001**	50.06	**<0.001**	21.07	**<0.001**
Total phosphorus accumulation	1615.47	**<0.001**	72.93	**<0.001**	9.80	**<0.001**

*The P values with significant differences were bolded.*

* means the interaction between nutrient levels and AMF.

### Growth and Biomass Allocation

**FIGURE 2:**
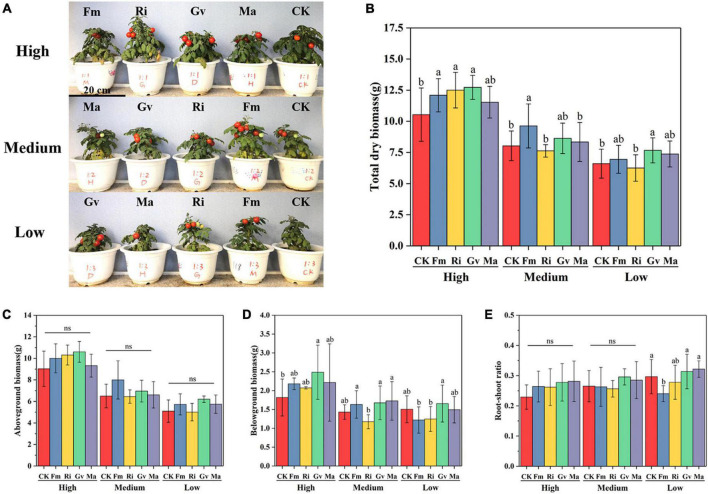
Growth **(A)**, total biomass **(B)**, aboveground biomass **(C)**, belowground biomass **(D)**, and root shoot ratio **(E)** of cherry tomato inoculated with different AMF treatments under different nutrient levels. ns denotes no significant difference between AMF inoculation treatments at the same nutrient level (*P* > 0.05), the same as below.

The aboveground and belowground biomass of cherry tomato was significantly affected by soil nutrient level and AMF inoculation treatment. The total biomass of cherry tomato was significantly affected by soil nutrient level, AMF, and their interactions. However, the root–shoot ratio was only significantly affected by AMF inoculation treatment (Table 2). The growth performance of cherry tomatoes under different AMF treatments were different under the three nutrient levels (Figure 2A). Aboveground biomass and root–shoot ratio did not change significantly after the four AMF inoculation treatments were applied. But the three single AMF treatments significantly increased the total biomass of cherry tomato, and Gv inoculation significantly increased the belowground biomass at a high nutrient level (Figures 2B–E). Fm inoculation could significantly increase the accumulation of total biomass, and the total biomass was significantly higher than that of Ri and Ma inoculation at the medium nutrient level (Figure 2B). AMF inoculation at medium nutrient levels did not significantly affect aboveground and belowground biomass and root–shoot ratio (Figures 2C–E). It is noteworthy that Ri inoculation significantly reduced belowground biomass compared to Fm, Gv, and Ma treatments (Figure 2D). When the nutrient level was lowest, aboveground and belowground biomass and root–shoot ratio did not change significantly after inoculation with four AMF treatments, while inoculation with Gv significantly promoted total biomass accumulation (Figures 2B–E).

### Reproductive Characters

**FIGURE 3:**
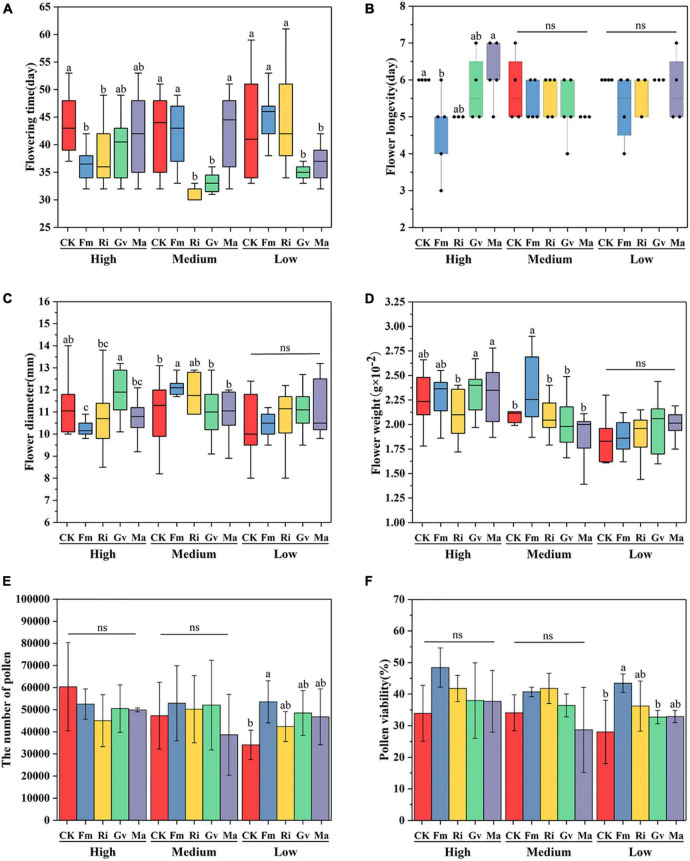
Flowering time **(A)**, single flower longevity **(B)**, single flower diameter **(C)**, single flower weight **(D)**, pollen quantity **(E)**, and pollen viability **(F)** of cherry tomato inoculated with AMF under different nutrient levels.

AMF inoculation and the interaction between AMF inoculation and nutrient level can significantly affect the flowering time of cherry tomato (Table 2). The flowering time of cherry tomato was significantly advanced by inoculating Fm and Ri at a high nutrient level, significantly advanced by inoculating Ri and Gv at a medium nutrient level and significantly promoted by Gv and Ma at a low nutrient level (Figure 3A). Nutrient levels, AMF inoculation, and their interactions had no significant effect on single flower longevity (Table 2). The single flower longevity of cherry tomato decreased after Fm inoculation at high nutrient levels (Figure 3B). The fresh weight and diameter of a single flower of cherry tomato were significantly different under different nutrient levels and the interaction between nutrient levels and AMF (Table 2). Inoculating Fm significantly reduced the flower diameter at high nutrient levels. At the medium nutrient level, inoculating Fm significantly increased the flower diameter and flower weight, while inoculating Ri significantly reduced the fresh flower weight of cherry tomato. When the nutrient level is at the lowest level, AMF inoculation treatment has no significant effect on flower diameter and flower weight (Figures 3C,D). AMF inoculation did not affect the pollen number and pollen viability of cherry tomato at high and medium nutrient levels, whereas Fm inoculation significantly increased the pollen number and pollen viability at low nutrient levels (Figures 3E,F).

**FIGURE 4:**
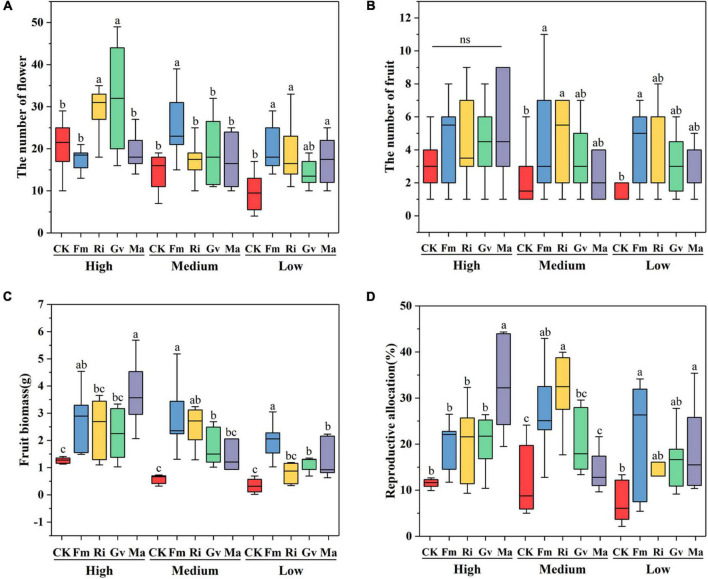
Effects of AMF inoculation on flower number **(A)**, fruit number **(B)**, fruit biomass **(C)**, and reproductive allocation **(D)** of cherry tomato under different nutrient levels.

Soil nutrient level, AMF, and their interaction had significant effects on flower number, fruit number, fruit biomass, and reproductive allocation of cherry tomato (Table 2). Ri and Gv inoculation significantly increased the flower number of cherry tomato under high nutrient levels. There were no significant differences in the fruit number of cherry tomato among the four AMF inoculation treatments under high nutrient levels. When the cherry tomato was grown at a medium nutrient level, Fm inoculation significantly increased its flower number, and Fm and Ri inoculation also significantly increased the fruit number. Fm inoculation treatment increased the number of flowers and fruits, but Fm, Ri, and Ma inoculation treatment only increased the flower number of cherry tomatoes at the lowest nutrient level (Figures 4A,B). Fm inoculation could increase the fruit biomass of cherry tomato at all three nutrient levels, but Ri inoculation only significantly increased the fruit biomass at the medium nutrient level. Gv inoculation could promote the increase of fruit biomass at medium and low nutrient levels, while Ma inoculation increased the fruit biomass of cherry tomato at high and low nutrient levels (Figure 4C). Fm and Ri inoculation at the medium and lower nutrient levels significantly increased the reproductive allocation, while Ma inoculation promoted resources allocation to the reproductive organs under a high nutrient level (Figure 4D).

**FIGURE 5:**
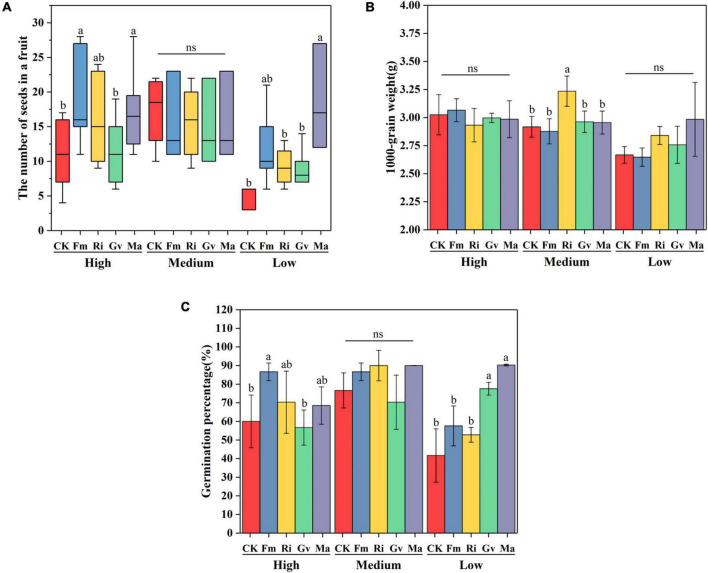
Effects of AMF inoculation on seed number in a fruit **(A)**, seed 1,000-grain weight **(B)**, and seed germination percentage **(C)** of cherry tomato under different nutrient levels.

Soil nutrient level and AMF inoculation had significant effects on seed number per fruit of cherry tomato, while 1,000-grain weight of seeds was only affected by soil nutrient level (Table 2). Fm and Ma inoculation significantly increased the number of seeds per fruit but had no effects on the seed 1,000-grain weight at a high nutrient level. The four AMF inoculation treatments had no significant effects on the seed number per fruit, while Ri inoculation significantly increased the seed weight at the medium nutrient level. Ma inoculation tended to increase the seed number per fruit but did not affect seed weight at low nutrient levels (Figures 5A,B). Seed germination percentage was significantly affected by soil nutrient levels, AMF inoculation, and their interactions (Table 2). Seed germination percentage could be improved by inoculating Fm at a high nutrient level and by inoculating Gv and Ma under low nutrient levels (Figure 5C).

### Accumulation of Nitrogen and Phosphorus

**FIGURE 6:**
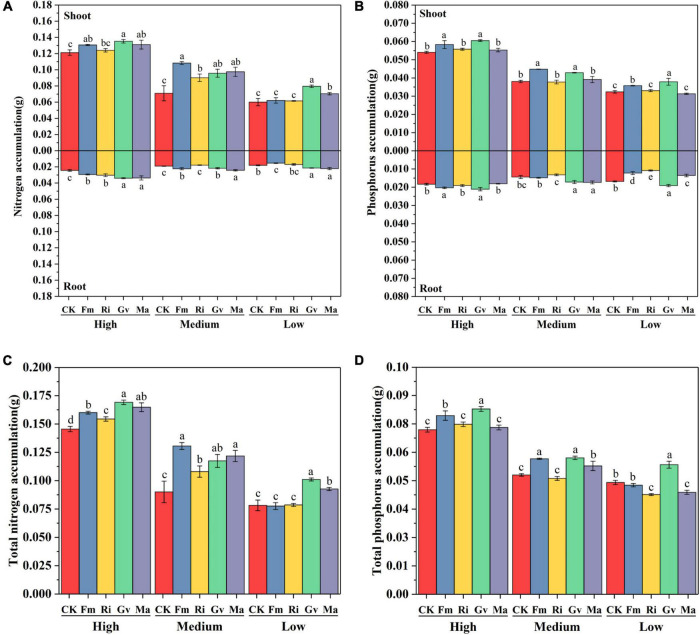
Effects of AMF inoculation on nitrogen accumulation **(A)**, phosphorus accumulation **(B)**, total nitrogen accumulation **(C)**, and total phosphorus accumulation **(D)** of aboveground and belowground cherry tomato under different nutrient levels.

The accumulation of nitrogen and phosphorus in cherry tomato was significantly affected by soil nutrient level, AMF inoculation, and their interaction (Table 2). Fm, Gv, and Ma inoculation promoted the aboveground nitrogen accumulation of cherry tomato. The four AMF inoculation treatments all increased the belowground and total nitrogen accumulation of cherry tomato under high nutrient levels. Four AMF inoculation treatments significantly increased the aboveground and total nitrogen accumulation of cherry tomato under medium nutrient level, while Fm, Gv, and Ma inoculation treatments increased the belowground nitrogen accumulation of cherry tomato. When cherry tomato was grown under low nutrient level, inoculation of Gv and Ma significantly increased the aboveground nitrogen accumulation, belowground nitrogen accumulation, and total nitrogen accumulation of cherry tomato (Figures 6A,C). In addition, Fm and Gv inoculation at the three nutrient levels all significantly increased the aboveground phosphorus accumulation of cherry tomato. The effects of different AMF inoculation on belowground phosphorus accumulation were different under different nutrient conditions. Fm and Gv inoculation under high nutrient levels can increase the belowground phosphorus accumulation. Gv and Ma inoculation at a medium nutrient level and Gv inoculation at a low nutrient level significantly promoted the belowground phosphorus accumulation and total phosphorus accumulation. Ri inoculation at medium and low nutrient levels decreased the belowground phosphorus accumulation of cherry tomato. Fm and Ma inoculation also significantly reduced the belowground phosphorus accumulation under low nutrient levels, and inoculation of Ri and Ma under low nutrient levels was not conducive to total phosphorus accumulation in cherry tomatoes (Figures 6B,D).

## Discussion

### Effects of Nutrient Conditions on Arbuscular Mycorrhizal Fungi Infection and Growth Promotion

Although AMF colonization on cherry tomato roots was high (41.15–71.30%), our study did not find that AMF inoculation at a high nutrient level reduced the dependence of cherry tomato on AMF. It was found that the dependence of cherry tomato on AMF was stronger at a higher nutrient level. With the decrease of nutrient level, the promoting effect of AMF decreased to a certain extent (Figure 2B), which indicated that the high nutrient level in this experiment was not enough to inhibit the colonization of AMF and its contribution to cherry tomato. Our study results showed that the four AMF inoculation treatments showed significant differences in affecting the growth and reproduction of cherry tomato. On the one hand, the accumulation of plant biomass depends on the symbiotic effect of AMF, and on the other hand, it is also affected by soil nutrients. There seems to be a positive correlation between infection rate and biomass (Ma et al., 2007). Different AM fungi in soil with different nutrient levels may have different mechanisms in promoting plant growth and development. Currently, it is generally accepted that AMF can regulate the balance of plant hormones and thus participate in promoting plant growth, especially play an important role in the regulation of auxin (IAA), gibberellin (GA), cytokinin, ethylene, and other plant hormones and signaling substances (Pozo et al., 2015; Shi et al., 2015). Furthermore, a study has found that AMF can improve the activity of photosynthesis key enzyme (ribulose bisphosphate carboxylase, RuBisCO), and thus increase the net photosynthetic rate of leaf, which facilitated the accumulation of photosynthates (Pozo et al., 2015). However, the effect of enhancing photosynthesis is also restricted by AMF species, which indicated that AMF have different functions under different nutrient conditions.

### Effects of Arbuscular Mycorrhizal Fungi on Reproductive Characters of Cherry Tomato Under Different Nutrient Conditions

Our study confirmed that the inoculation of AMF could advance the flowering of cherry tomatoes, but this advanced effect is limited by AMF species. The earlier flowering of plants is mainly promoted by a large amount of phosphorus provided to the host plant after AMF inoculation (Oehl et al., 2011; Kazadi et al., 2021). However, this is not the only reason. According to the C/N ratio theory, the formation of flower organs in plants is controlled by the ratio of sugar content to nitrogen content (C/N) in plants (Kraus and Kraybili, 1918). Because photosynthesis is also affected by nitrogen content (Evans, 1983), the nitrogen supply provided by AMF after symbiosis with plants can also promote the flowering of plants, which is consistent with the results of nitrogen accumulation. On the other hand, flower bud differentiation is also affected by hormone levels and effective assimilate interactions in plants. Some AMF can promote the uptake of potassium in plants, and increase the synthesis of auxin (IAA) and gibberellin (GA) in plants, thus inducing flower bud formation and flowering earlier (Asmelash et al., 2016; Liu et al., 2018). Flower diameter and weight seem to be affected only under medium nutrient levels inoculated by *F. mosseae*, while under the low nutrient level, inoculation with *F. mosseae* significantly promoted pollen viability and pollen number of cherry tomato. This effect may be because AMF provides more phosphorus and potassium to plants and thus increases plant investment in floral organs under the low nutrient level. It is well known that the formation of flower and pollen require phosphorus and potassium. AMF may contribute to flower production by providing both phosphorus and potassium to plants during flowering (Conversa et al., 2015; Pereyra et al., 2019). Increasing pollen number and viability under low nutrient levels may also be a reproductive strategy for plants to symbiosis with AMF when nutrients are scarce, which may be more conducive to the continuation of plant offspring under nutrient deficiency conditions.

We also found that inoculation of AMF has a positive effect on the flower and fruit set of cherry tomato. Especially at low nutrient levels, the promotion effect of AMF inoculation on flower numbers was more obvious. The result of this positive effect was that cherry tomatoes may have more fruit and increased the biomass of the fruit, which was also proved in our study (Figure 4). For most flowering plants, the number of flowers is proportional to the biomass due to resource restriction. Phosphorus is one of the most important limiting factors. The symbiosis of AMF can ameliorate the restriction of resources, especially phosphorus, on plant growth. When nutrients are relatively scarce, AMF inoculation can increase the number of flowers of tomato (David and Roger, 1990; Püschel et al., 2014). Moreover, the increase in flower number may also be caused by the increased concentration of photosynthetic products and plant hormones regulated by AMF (Bona et al., 2017). In particular, the increase of the content of gibberellin (GA) in plants can make plants produce more flowers (Bona et al., 2015). We found that the number of cherry tomato fruits did not seem to be directly affected by the number of flowers, although the number of flowers was a major factor affecting the number of cherry tomato fruits. In this study, the increase in flower numbers of the cherry tomato did not directly increase fruit numbers under high nutrient conditions. In addition, the effects of different AMF inoculation on the number of cherry tomato flowers and fruits were also different under different nutrient conditions. The fruit develops from the ovary or together with other parts of the flower. The growth of the ovary relies mainly on a large number of hormones and nutrients. The phosphate and the production of regulating hormones provided by AMF after symbiosis can promote the growth and development of the ovary (Zhang et al., 2014; Hart et al., 2015; Battini et al., 2016; Bona et al., 2017). Nevertheless, the effects of different nutrient levels on cherry tomato reproduction under different AMF inoculation treatments were different. This suggests that changes in fruit yield may be an adaptive strategy for cherry tomato to tradeoff between AMF infection and soil nutrient resource restriction.

### Effects of Arbuscular Mycorrhizal Fungi on the Reproductive Allocation of Cherry Tomato Under Different Nutrient Conditions

We found that inoculation of single AMF species did not seem to promote the reproductive investment of cherry tomato at a high nutrient level. But the reproductive allocation of cherry tomato increased after being inoculated with the mixed AMF species. It suggests that communities containing multiple AMF species may jointly promote the reproductive investment of cherry tomato under natural conditions. Our results also confirmed that the mixed AMF species inoculation treatment (Ma) also increased the reproductive allocation at the low nutrient level. The results indicated that the extracellular mycelia formed after the infection of cherry tomato with multiple AMF greatly increased the decomposition and utilization efficiency of unavailable nutrients in the soil, thus increasing the uptake of nitrogen and phosphorus by cherry tomato and increasing the resource investment in reproduction (Barrett et al., 2011; Schnepf et al., 2011; Zhang et al., 2014). For single AMF inoculation, the effect of *F. mosseae* was the most prominent, which significantly increased the reproductive allocation of cherry tomato at medium and low nutrient levels. This also fully indicated that when the growth and reproduction of cherry tomato were stressed by nutrients, the functions of different AMF inoculation treatments were different. *F. mosseae* had a stronger ability to relieve such stress, which can promote the increase of reproductive allocation and improve the reproductive success of cherry tomato.

In our study, we found that mixed AMF inoculations (Ma) increased seed number in two extreme soil environments (high and low nutrient), which also appears to be phosphorus-driven (Bona et al., 2017). However, it is very interesting to note that although inoculation with *R. intraradices* at the medium nutrient level did not affect the cherry tomato seed number, it did affect the weight of the seeds. This suggests that AMF inoculation may increase the reproductive allocation of the host plant by putting more resources into offspring seed (such as increasing seed diameter and nutrient content) (Liu et al., 2018). The germination percentage of cherry tomato seeds increased after AMF inoculation at a low nutrient level, because the seed germination process was affected by endogenous hormone levels and organic matter accumulation (Li et al., 2012; Han and Yang, 2015). The improvement of seed germination percentage can prove that AMF can promote plant resource investment in seeds, which indicates that AMF may improve the ability of plants to sexually reproduce in an adverse environment. Therefore, the effects of the same/different AMF inoculation under different nutrient conditions are also different, which may be driven by the context-dependency of AMF–plant symbiosis (such as phosphorus content or phosphorus–nitrogen ratio) or influenced by the functional differences of AMF under heterogeneous soil conditions (Johnson et al., 2015).

### Effects of Arbuscular Mycorrhizal Fungi on Nitrogen and Phosphorus Accumulation of Cherry Tomato Under Different Nutrient Conditions

Our study found that AMF enhanced total nitrogen accumulation of cherry tomato at high and medium nutrient levels, while at a low nutrient level, promotion effects of AMF inoculation excluding *F. mosseae* on total nitrogen accumulation seemed to disappear. Cherry tomato inoculated with *F. mosseae* reduced root nitrogen accumulation at a low nutrient level. Different AMF species showed a great difference in influencing phosphorus accumulation. We found that *F. mosseae* and *G. versiforme* had a significant promotion effect on the accumulation of phosphorus in the aboveground of cherry tomato. When cherry tomato was inoculated with AMF under low nutrient level, some AMF inhibited the absorption and accumulation of phosphorus in the root system and decreased the total accumulation of phosphorus. The allocation pattern of nutrients in the aboveground and belowground of plants is a concrete embodiment of the survival strategy of maximizing the utilization of plant resources. The infection of AMF on cherry tomato changed nutrient allocation patterns in aboveground and belowground parts. AMF extracellular mycelium can accelerate the mineralization of organic phosphorus and the dissociation of hard-to-melt phosphorus in the soil, making it easier for plants to absorb. AMF extracellular mycelium also can promote the absorption of ammonia nitrogen and nitrate nitrogen by plants (Leigh et al., 2009; Schnepf et al., 2011). The effect of AMF on plant nutrient absorption was affected by soil nutrient differences and showed different promoting effects. When nutrient conditions were good, plants could absorb nutrients together through roots and mycelia, while in a nutrient-deficient environment, the presence of AMF increased the belowground energy consumption of plants, resulting in a decrease in the ability of plants to accumulate nutrients. Our results indicate that functional differences of AMF lead to variations in the nutrient accumulation in cherry tomato, and such variations are also affected by nutrient levels.

## Conclusion

This study showed that cherry tomato growth and reproduction were closely related to soil nutrient content and AM symbiosis. The function of AM symbiosis also varies with soil nutrient levels. We found that AM symbiosis had direct promoting effects on the growth and nutrient accumulation of cherry tomato, and confirmed that AM symbiosis promoted flower and seed setting of cherry tomato. In addition, the phenotypic response of the growth and reproduction of cherry tomato to mycorrhizal symbiosis under different nutrient conditions was revealed, and the promoting effects of different AM fungi on cherry tomato growth were found to be different under heterogeneous conditions. It proved that different nutrient levels in soil determined the difference of cherry tomato’s benefits from mycorrhizal symbiosis.

## Data Availability Statement

The original contributions presented in the study are included in the article/Supplementary Material, further inquiries can be directed to the corresponding author.

## Author Contributions

LW: experiment, data curation, methodology, visualization, writing-original draft, writing-review, and editing. XC, YD, and DZ: experiment and data curation. ZT: funding acquisition, supervision, resources, review, and editing. All authors contributed to the article and approved the submitted version.

## Conflict of Interest

The authors declare that the research was conducted in the absence of any commercial or financial relationships that could be construed as a potential conflict of interest.

## Publisher’s Note

All claims expressed in this article are solely those of the authors and do not necessarily represent those of their affiliated organizations, or those of the publisher, the editors and the reviewers. Any product that may be evaluated in this article, or claim that may be made by its manufacturer, is not guaranteed or endorsed by the publisher.
