# Supplementary material for: Nutrients Regulate the Effects of Arbuscular Mycorrhizal Fungi on the Growth and Reproduction of Cherry Tomato
Source: Front Microbiol. 2022 Apr 8;13:843010. doi: 10.3389/fmicb.2022.843010 (PMC9024412; doi:10.3389/fmicb.2022.843010)
Supplement: Supplementary file 1 [file Data_Sheet_1.DOCX]

**Support materials 1**: Culture of AMF strains

**1 Propagation of AMF strains**

**1.1 Commercial mycorrhizal strains**

The AMF strains used in this study was purchased from “Bank of Glomeromycota in China, BGC” at Institute of Plant Nutrition, Resources and Environment, Beijing Academy of Agriculture and Forestry Sciences.

**Table 1. The AMF species and BGC number used in this study.**

| **AMF species** | **BGC number** | **National Microbial Resource Center ID(China)** | **Host plant** |
| --- | --- | --- | --- |
| *Funneliformis mosseae* | BGC HEB02 | 1511C0001BGCAM0063 | *Zea mays* L. |
| *Rhizophagus intraradices* | BGC HEB07D | 1511C0001BGCAM0056 | *Lycopersicon esculentum* and *Cucumis sativus* L. |
| *Glomus versiforme* | BGC NM03C | 1511C0001BGCAM0032 | *Medicago sativa* |

**1.2 Propagation and culture of AMF strains**

**Preparation of culture substrate:** After natural air drying, the clean sand after washing was put into bags and sterilized in high temperature steam sterilization at 121°C /120min. The sterilization condition was repeated twice with an interval of 3 days. After sterilization, it was cooled naturally for later use.

**Instrument disinfection:** During the propagation process, paper cups, scissors and sieves used are wiped and disinfected with 95% ethanol solution to avoid contamination by other fungal contaminants.

**Seed disinfection and germination:** The corn (*Zea mays* L.) and *Trifolium repens* L. seeds used in the propagation process of AMF strains culture need to be sterilized on the surface. All seeds are soaked in 0.5% potassium permanganate for 5 min, 10% H_2_O_2_ for 10 min and 5% NaClO_3_ for 1 min respectively. Finally, the seeds are washed with pure water and then placed in petri dishes with moist filter paper. The petri dishes with seeds were placed in a constant temperature light incubator at 25°C (20% illumination and 60% humidity) for germination, and AMF inoculation and culture could be carried out after the seeds were blanched.

**Preparation of Hoagland Nutrient Solution:** Hoagland nutrient solution reagents used in this study was purchased from Qingdao Hope Bio-Technology Co., Ltd. (Product number: HB8870-1). Nutrient solution components are as follows: Calcium salt: 945 mg/L; Potassium sulfate: 607 mg/L; Ammonium dihydrogen phosphate: 115 mg/L; Magnesium sulfate: 493 mg/L; Sodium ferric EDTA: 20 mg/L; Ferrous sulfate: 15 mg/L; Borax: 4.5mg /L; Boric acid: 2.86 mg/L; Manganese sulfate: 2.13 mg/L; Copper sulfate: 0.05 mg/L; Zinc sulfate: 0.22 mg/L; Ammonium sulfate: 0.02 mg/L.

**Greenhouse culture conditions:** The photoperiod of the greenhouse is 12 h/12 h(light/dark), the optical quantum flux density is 550-600 μmol/m^2^s, the temperature is 30/25°C(day/night), and the humidity is 40-50%.

**Mycorrhizal inoculation:** Fill the paper cup with sterilized clean sand culture matrix to 2/3 of the paper cup, and add about 10g of AMF bacteria agent (including spores, infected root segments and mycelia) containing AMF spores determined after the above inspection into the center of the paper cup, then fill the surrounding with sterilized river sand culture matrix, and pour in pure water. Sow 1 corn seed/pot and 30-50 *T. repens* L. seeds in cup, cover the surface with 0.5 cm sterilized matrix, then move to the greenhouse for culture, water every 2 days to ensure the normal growth of plants, and avoid excessive water application, apply Hoagland Nutrient Solution every 2 weeks, the culture cycle is 4 months.

**Acquisition of AMF strain:** After the culture of AMF strain, we stopped watering into the soil of paper cups. The plants were harvested and naturally dried, the shoots of plants were cut using scissors. AMF propagation matrix and plant roots were mixed together, and then sieved by the 100-mesh sieve. The roots left in the sieve were cut into 0.3~0.5 cm root segments using scissors. Then, root segments were added into the matrix after sieving and mixed evenly to obtain the AMF strain required in this experiment.

**1.3 Examination of spore density in AMF strain**

AM fungal spores being isolated and extracted by wet sieve declination.

Firstly, clean sieve with pore sizes of 0.88 mm, 0.25 mm and 0.055 mm were stacked together and the pore sizes gradually increased from bottom to top. Then, weigh 20 g soil samples into a clean beaker, add tap water to soak the soil samples, stir with a clean glass stick, mix thoroughly and let suspension stand for 30 s. Slowly pour the supernatant into the top sieve and strain. Rinse the soil sample in the beaker repeatedly with running water until the supernatant becomes clear. Rinse the sieve layer by layer, collect the debris from the middle and bottom sieve into 50ml centrifuge tube, then centrifuge at 2000 rpm for 3 min, discard supernatant, add 50% sucrose solution, centrifuge again at 2000 rpm for 3 min, take out centrifuge tube. Pour the supernatant into a clean 0.055mm soil sieve and rinse 2-3 times with running water. Finally, the washed spores and debris were put into petri dishes and counted under light microscope.


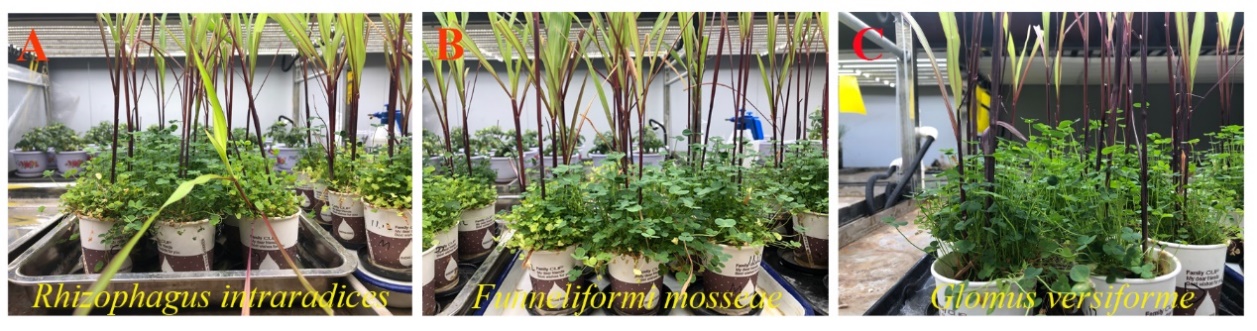


**Figure 1. Greenhouse culture of three AMF strains. A：*Rhizophagus intraradices*；B：*Funneliformi Mosseae*；C：*Glomus versiforme*.**
